# The Work of Hospital Almoners

**Published:** 1917-07-14

**Authors:** 


### THE MATRONS' AND SISTERS' DEPARTMENT.

### THE WORK OF HOSPITAL ALMONERS.

### Address by Miss Cummins at the London School of Economics.

THE work of hospital almoners is little known even in London, which was its birthplace, and it is a matter of satisfaction to the Hospital Almoners' Council that the subject was selected by the London School of Economics as worthy of your attention this afternoon at a time such as this, when the field of activities for social workers is increasing so rapidly.

I repeat, the work of hospital almoners is very little known, and is generally summed up as "stopping hospital abuse." Well, it is certainly a fact that at many hospitals an almoner was first appointed with an idea of preventing the abuse which is apt to occur in an almost chaotically large city such as London. Years ago it was no very unusual occurrence for well-to-do patients, living in comfort if not in affluence, to come to the London hospitals for free treatment. In my own experience I could quote patients, often with quite minor ailments, owning their motor-cars or having very large incomes, who have applied for treatment; and there is no doubt that the careful inquiries into the circumstances of each patient instituted by the almoners' department has to a large extent prevented this form of abuse.

Possibly one case may be quoted as an example. Mrs. X, a married woman, respectable-looking, very plainly dressed, stated that her husband never earned more than £2 a week, and that she had great difficulty in paying expenses owing to illness. She came to St. Thomas's because a year ago her private doctor had arranged for her to be operated upon by a member of the staff, and she said that she could no longer afford to pay for medical advice. She was very reluctant to give her doctor's name, and refused his address. Treatment was deferred pending the result of inquiries, and her doctor, who was traced, wrote: "It is nothing short of scandalous that this lady should seek for free treatment and advice. Her husband has just bought a house and paid £700 down; they own their late house, which lets at £50. They both have a fair amount invested in stocks and shares. Mr. X is manager of a very old-established firm of pawnbrokers."

Such cases are not very frequent, but you will agree, I know, that charitable funds should not be expended on them, and that the boards of management of hospitals are justified in establishing some sort of supervision to prevent the expenditure both of hospital funds and the time and skill of the hospital staff on those who are in a position to pay for their own treatment.

Next we find a large number of insured patients who come up to the hospital for minor ailments. Some come because they want a change from their panel doctor, or fancy that they get better medicine at the hospital, and some because their panel doctor wants a change from them! Unless these patients need some specialised form of treatment, they are referred back to their panel doctor, after medical examination, as they are not suitable for medical charity.

Then we come to the third and most difficult class. Amongst the patients who daily present themselves for treatment there are always a few who, sick and ill and in obvious need of medical treatment, are in such a destitute and unsatisfactory condition that to grant out-patient treatment would be totally inadequate. These cases are extremely difficult to deal with, and are rarely refused on first attendance unless they are already in receipt of outdoor relief. They are allowed, perhaps, to attend two or three times while careful inquiry is made, but if it is found impossible to improve their conditions they are eventually referred to the Poor-Law authorities, as it is found that attendance at the hospital is of no benefit to them and that therefore it would be misuse of charitable funds to allow them to give them hospital treatment.

But if an almoner's work consisted in this form of semi-negative work—useful and right as it doubtless is—I doubt if it would have attracted the band of workers who are now engaged in it. In America, where there are State hospitals, and where I am told that the question of abuse hardly existed—in most of the big hospitals there are social service departments, where educated women carry on practically the same work as our hospital almoners, and where there are training centres for candidates who are anxious to undertake similar posts. I have had visits from hospital social workers from the States, and have been immensely interested in hearing how exactly the same problems, though in different surroundings, present themselves in American hospitals, and am deeply indebted to many American workers for inspiration and suggestions.

To an audience such as this it is quite unnecessary for me to dwell on the fact that social and physical conditions are closely interwoven. All here are well aware of the ever-present problem of whether poverty causes disease or disease causes poverty.

Thus we find the social worker, when trying to improve the home condition of a child, sending its father up to the hospital to ask the physician whether there is any physical reason to account for the man's apparent laziness; or the physician turning to the social worker to find out whether social conditions are impeding or retarding his patient's recovery.

For the time has happily long gone by when a bottle of medicine could be considered sufficient treatment for most ailments, even though many of the poorer patients still cling to the bottle of hospital medicine as a panacea for all evil, and still one of our most difficult tasks is to teach them that medicine alone will not cure them, and that they must try to carry out all the instructions of the doctor and thus co-operate in their own treatment. That many of the patients are quite unable to do this without help will, I know, be readily accepted, and it is on this that much of the almoner's work is centred.

The almoner, therefore, has first to decide as to the suitability of the patient for hospital treatment, and here I should like to emphasise two points :—

(1) That all patients go first to the doctor for examination before seeing the almoner. He or she may be in urgent need of immediate treatment which cannot be delayed, and, furthermore, until the doctor has diagnosed the case it is impossible to decide whether or no a patient can afford to provide his own treatment. A father, who can easily afford to pay for some minor ailment for his child, may equally be totally unable to meet the expenses of a long illness.

(2) That no patient is refused treatment without the consent of the doctor. It may be a case that he considers should be seen several times before giving an opinion, or it may be a case that is of great clinical interest. As a rule, the doctor is quite willing to allow the matter to be judged on social grounds alone, but, in the best interests of the patient, the almoner, and the hospital, the final decision rests with the doctor.

This, then, is the part of the almoner's work to which I referred as being partially of a negative character, although its value in the development of independence and self-help cannot be overlooked.

But the work only begins there. Having decided that the patient is a suitable one, it is the almoner's duty to ensure that he or she is able as far as possible to carry out the doctor's directions effectively. (1) The mother may be told to give the child plenty of milk; (2) the man is advised to get artificial teeth; (3) the girl is instructed to give up factory work, get a rest in the country, and have plenty of nourishing food. But it may be that all these instructions are equally impossible unless there is someone at hand to plan and advise, and possibly direct the patient to the right channel for assistance.

For this the almoner's work is based on co-operation. It has been said that the almoner is in the hospital partly to act as a reminder that doctors and social workers, hospitals and charitable agencies, can only do their best work in combination. I repeat that it is for the almoner to ascertain if the patient can carry out the doctor's instructions.

If the man can pay for his artificial teeth himself, if the mother can afford the daily pint or quart of milk, if the factory girl can get a holiday and has friends in the country who will take her, well and good; the almoner has only to see that it is done. But unfortunately, as you will agree, a very large proportion of the patients do not even contemplate such possibilities. Sometimes, after a long and intimate talk, one is able to form some plan by which the doctor's orders may be carried out. A letter to the man's employer may bring a promise of help or a loan from the firm. The grandmother may possibly be able to subscribe to the baby's milk, or the father may be seen after work-hours and consent to contribute rather more largely to the housekeeping. But it is far more likely that the cases may have to be directed to some organisation outside the hospital, and a letter sent to its secretary explaining the doctor's instructions. At St. Thomas's Hospital last year 7,616 patients were referred to charitable agencies, either for visiting and watching or for relief, in many cases the almoner acting only as a connecting link or directing centre. *(To be continued.)*

